# Double-Bind of Recruitment of Older Adults Into Studies of Successful Aging via Assistive Information and Communication Technologies: Mapping Review

**DOI:** 10.2196/43564

**Published:** 2022-12-23

**Authors:** Najmeh Khalili-Mahani, Kim Sawchuk

**Affiliations:** 1 Media-Health Lab Milieux Institute for Arts, Culture and Technology Concordia University Montreal, QC Canada; 2 Culture and Mental Health Research Unit Lady Davis Institute Jewish General Hospital Montreal, QC Canada; 3 McGill Centre for Integrative Neuroscience Montreal Neurological Institute McGill University Montreal, QC Canada; 4 Ageing, Communication and Technology Lab Department of Communication Studies Concordia University Montreal, QC Canada

**Keywords:** information and communication technologies, successful aging, healthy aging, independent living, agism, research methods, double-bind theory, mobile phone

## Abstract

**Background:**

Two fields of research and development targeting the needs of the aging population of the world are flourishing, *successful aging* and *assistive information and communication technologies* (A-ICTs). The risks of ageist stereotypes emerging from how we communicate in both discourses are long known. This raises questions about whether using specific age criteria in the context of “aging deficits” can bias participation in, or compliance with, the research process by older adults who try to avoid age-related stigma.

**Objective:**

This study aimed to examine subject recruitment, study designs (based on age >65 years criteria), as well as discourses in research objectives and conclusions in health research on affordances of A-ICTs for older adults.

**Methods:**

A systematic mapping approach was used to characterize rationales, methods, stated objectives, and expected outcomes of studies indexed in PubMed and retrieved through the search logic ([“Older Adults” OR Seniors OR Elderly] AND [ICT OR gerontechnology OR “Assistive Technology”)] AND (“Healthy Aging” OR “Successful Aging” OR “healthy ageing” OR “successful ageing”). Inclusion criteria were as follows: the study should have recruited older participants (aged >65 years), been qualitative or quantitative research, and involved the introduction of at least one A-ICT for health-related improvements. Exclusion criteria were as follows: reviews, viewpoints, surveys, or studies that used information and communication technology for data collection instead of lifestyle interventions. Content, thematic, and discourse analyses were used to map the study characteristics and synthesize results with respect to the research question.

**Results:**

Of 180 studies that passed the search logic, 31 (17.2%) satisfied the inclusion criteria (6 randomized controlled trials, 4 purely quantitative studies, 9 focus groups, 2 observational studies, and 10 mixed methods studies). In all but one case, recruitment was pragmatic and nonrandom. Thematic analysis of rationales revealed a high likelihood of emphasis on the burdens of aging, such as rising costs of care (12/31, 39%) and age-related deficits (14/31, 45%). The objectives of the research fell under 4 categories: promotion of physical activity, acceptance and feasibility of robots and remote health monitoring systems, risk detection, and the future of A-ICTs in health care for older adults. Qualitative studies were more attentive to the nonageist research guidelines. Heterogeneity in the study results (both qualitative and quantitative) was not related to age but to individual agency, acceptance, and adherence. A combination of research strategies (participatory, longitudinal, playful, flexible, and need-based designs) proved successful in characterizing variations in study outcomes. Studies that documented recruitment dynamics revealed that fear of stigma was a factor that biased participants’ engagement.

**Conclusions:**

This review indicates that age is not an informative criterion for recruitment and retention of participants. Charting the dynamics of adoption of, and interaction with, A-ICTs is critical for advancing research and technology development.

## Introduction

### Background

Two fields of research and development that target the needs of the aging population of the world are flourishing: one field focuses on discovering pharmacological or behavioral solutions that promote successful or healthy aging [1-3]. The term successful aging (introduced in 1987 by Rowe and Khan) refers to the heterogeneity in health conditions among people of the same age [4]. These authors defined successful aging as different and superior to “usual ageing” (marked by a statistical likelihood of decline in physical and mental health). They listed several potential factors (ranging from biology to psychology, socioeconomic, and personal contexts) that would vary across people of the same age and determine whether they would retain full health and control in old age (successfully aged). According to the World Health Organization, healthy aging reflects the capacity to maintain functional abilities that support one to be mobile and active, meet basic needs, build and maintain relationships, contribute to society, and learn, grow, and make decisions [5].

Another field focuses on developing assistive information and communication technologies (A-ICTs) that help aging populations achieve the goal of healthy and independent living in later years of life [6,7]. A-ICTs are a component in digital health characterized by wireless and portable communication and computation technologies that make them “smart” and “social.” They are typically promoted as offering new opportunities for the extension of the (presumably declining) physical, cognitive, and social capacity of older adults to help them live safely and retain their independence in the course of aging [8].

In an empirical analysis of the discourses of successful aging associated with the use of sophisticated technology, in policy documents in Europe (from 2000 to 2021), Greubel et al [9] have shown that the discourse of “develop technology to do good” is dominant (ie, to address the unmet needs of a stereotypical older adult with certain physical and mental deficits). But in doing so, this discourse creates a “bad” aging stereotype: one who is incapable of or uninterested in adopting such technologies. Mort et al [10] have shown that if the discourse of aging with telecare is perceived as a coercive method in times of economic austerity (rather than true care), it can create resistance to the successful adoption of technologies that can indeed become useful for the aging population.

Given the heterogeneity of the aging process and the significance of the topic in both social and medical research, cultural and communication factors must be regarded in various stages of design, development, testing, knowledge mobilization, and policy making on these issues [9,11].

### The Double-Bind in Medicalized Age Research

The theory of double-bind by Gregory Bateson [12,13] helps explain the challenges of communicating with aging populations about their need for technology. Double-bind arises when the following conditions co-occur: (1) two or more individuals are involved in a relationship with high physical or psychological survival value for at least one of them (eg, older adults need to grow and their health care system is responsible for that); (2) in this relationship, messages are regularly given that, at one level of communication, assert something (eg, aging is not a deficit, and if it is, technology can overcome it), but at another level, negate or conflict with this assertion (eg, aging causes deficit, and deficits make technology uptake difficult); (3) messaging implies cost and punishment (eg, age-related deficits are costly, but costly technology can reduce them; if technology is not adopted, the risks and costs increase); and finally, (4) those in the relationship can neither escape the relationship nor are they allowed or able to comment on it (eg, no one can escape the reality of aging, nor can anyone stop technology innovation).

The consequence of such double binding in gerontology is ageism, a term coined by Niel Robert Butler (a geriatric physician and the director of the National Institute of Ageing), who expressed concern about stigmatizing older adults based on the prevalence of disease in older age [14].

The medicalization of aging originates from traditionally reductionist approaches to public health [15]. Numericizing age has been used as an index for political and socioeconomic agendas [16], for example, for predicting the mortality and insurance costs based on calculation of life expectancy [17]. Age also has a social meaning (in terms of life stages and roles, which are culture-dependent and can create both positive and negative age-related stereotypes). Medicalized approaches to aging, although important, create stereotypes [18-21]. Research indicates that industrial thinking about productivity and the workforce also contributes to negative stereotypes [22,23]. Culturally, these factors may create a worldview that leads to ageism [14,24-26] to such an extent that even studies about ageism risk themselves becoming ageist [27].

Medical studies on aging have long focused on the risks of research methods in producing or reproducing systemic ageist biases. In 1993, the Task Force to Develop Non-Ageist Guidelines for Research [28], sponsored by the American Psychology Association Board of Social and Ethical Responsibility and the Board of Scientific Affairs identified the following risks in studies designed to address the “problem of aging”: (1) confounding age with disease and disability based on the statistical assumption that the likelihood of certain dysfunctions was more prevalent in older adults; (2) using chronological age as an independent variable in cross-sectional studies of group (or intervention) differences; and (3) lack of attention to interactions among age, sex, and culture and the life-course patterns of individuals boxed in simplistic demographic classifications. The extent to which researchers are held to initial standards about nonageist research methods [28] or later guidelines by the American Psychological Association guidelines is not clear [29]. Guidelines such as the National Institute of Health’s Toolkit for Recruiting Older Adults into Research [30] emphasize the promotion of healthy (successful) aging. Culture also plays a significant role in the recruitment of older adults [31,32]. The question of how to overcome the double-bind of ageism and interest older adults in studies of A-ICTs is one of the motivations for the research in this study.

### Challenge of Age as a Recruitment Criterion in Health Technology Research

Research on health applications of information and communication technologies (ICTs) occurs at the intersection of medical sciences that treat aging as a preventable disease [33,34], technology that promises a solution to this problem [6,7], and humanities that are critical to stigmatization emerging from such discourse [9,10,35-38]. Age has a biological meaning (eg, in terms of developmental stages and longevity), but as a categorization criterion, what does it explain about the reality that 2 people of the same age may have entirely different medial and psychosocial experiences [4]?

In a social media study of older adults’ reactions to the media depiction of the needs of older adults for ICTs to cope with the stress of the pandemic, we noted a strong reaction to the implicit equivalence of “being old” with age >65 years, which is the retirement age in North America, particularly by those who considered themselves old but reminded the writers that their generation (eg, Bill Gates) pioneered information technologies [39]. This example highlights that using the age >65 years criteria in research involving ICTs could be problematic. Our research into the affordances of ICTs in improving the quality of life of older adults [39,40], suggests that concerns about “ageism” are on older adult’s mind. In addition, we have observed quantifiable differences in attitudes toward technology among those who participated in such studies and those who dropped out [41,42]. The motivating question in this study is, “Can focusing on *age-related deficits* as a reason for developing assistive technologies bias recruitment (based on 65+ criterion), and thus skew our understanding of the needs of older adults?”

Although many in their 60s are familiar with information technologies, recruitment and retention of older adults in studies that examine the affordances of A-ICTs is challenging because of the reality of an age-related digital division [43-45]. Several review studies have examined challenges and opportunities in the introduction of ICTs in providing health care to older adults, and they all emphasize that although such technologies are in principle promising and theoretically advantageous, there are important issues related to user acceptance, efficacy, and sustainable adoption that remain to be addressed (eg, see recent reviews in [46-49]). Among the general concerns expressed by older adults are fear of reducing the complexity of human experience with machine learning and artificial intelligence [50], fear of misinformation [51], fear of stigma [52,53], fear of surveillance [54,55], fear of losing control over their lives [56], and general technostress [39,57-60]. Of course, these concerns are not specific to older adults, and research suggests that there is a significant pushback against the notion of assumption that older adults are either unwilling or incapable of technology adoption [39,40,61,62].

The implication of ageism is self-exclusion from research [62,63]. Research suggests that data gathered about older adults are not entirely inclusive and that data collection methodologies bias inferences made about older adults [64,65]. Research shows that perceived ageism contributes to reluctance to engage in technology [44,45]. It is also plausible that those who experience the consequences of ageism (eg, depression and poor health conditions, as shown in this survey [38]) would be less likely to engage or participate in studies on this topic. Could the conceptual frameworks for linking A-ICTs and successful aging alienate participants who perceive the research to be stigmatizing them based on age?

### Research Objectives

The motivating question in this study is, “Can focusing on *age-related deficits* as a reason for developing assistive technologies bias recruitment (based on 65+ criterion), and thus skew our understanding of the needs of older adults?” In this study, we performed a mapping review to investigate medical research at the intersection of healthy aging and A-ICT to address the following questions:

1. What types of research methods and recruitment strategies are used in this line of research?
2. Which discourses and objectives drive the study rationales and objectives?
3. Are the study elements communicated in compliance with the Non-Ageist Guidelines for Research [28]?
4. Is age as a selection criterion informative?
5. Which research strategies are used to avoid age-related stereotypes?

## Methods

### Mapping Review

A mapping review framework was selected to classify and categorize information within the existing literature [66]. A mapping review does not have any preconceived plans for evaluating specific outcomes (as systematic reviews do) or any specific research question or intervention (as scoping reviews do). Instead, it relies on a sampling frame for a general topic and integrates data (qualitative or quantitative) to formulate questions for future systematic reviews [66]. The steps taken to conduct the review have been described in the sections below.

### Selection of Relevant Sources

As we were interested in the intersection between medical research, technology, and successful aging, we conducted our research in PubMed only. We searched for any articles satisfying the logic: ([“Older Adults” OR Seniors OR Elderly] AND [ICT OR gerontechnology OR “Assistive Technology”]) AND (“Healthy Aging” OR “Successful Aging” OR “healthy ageing” OR “successful ageing”).

### Inclusion and Exclusion Criteria

To be included in the review, studies had to satisfy the following criterion:

- Used age as a numerical selection variable
- Involved a quantitative or qualitative research component, in which older adults were recruited as participants
- Introduced ICTs to enhance the experiences of an exclusively aging population

Studies were excluded if they were reviews, surveys, viewpoints, or used ICTs for any purpose other than assisting users (eg, cases in which ICTs were used for simulation or data collection).

### Classifying, Categorizing, and Mapping

Articles that met the selection criteria were reviewed to retrieve the following information: study design and recruitment strategy (from *Methods* sections); research contexts and stated objectives (from *Introduction*—usually the first paragraph in an article that situated the work in relation to the needs of older adults, followed by a rationale paragraph that contextualizes the objective of the research presented in the article), and outcomes and conclusions (from *Discussion* and *Limitations* sections).

Content analysis was performed to assess the prevalence of research methods. Next, we performed a thematic analysis of expected research outcomes. All papers were read and open-coded for the specific outcomes expected from the intervention, based on the results and discussion section of the paper. These codes were then collapsed to identify the specific outcome categories related to our research questions.

### Synthesizing Results to Answer Research Questions

Having mapped the general characteristics of research studies within the review, we reread articles within each outcome category and performed a discourse analysis to answer our primary research questions and identify research strategies that mitigate implicit biases arising from the double-binding reality of *successful aging*.

## Results

### Selection of Sources of Evidence

#### Overview

**Figure 1:**
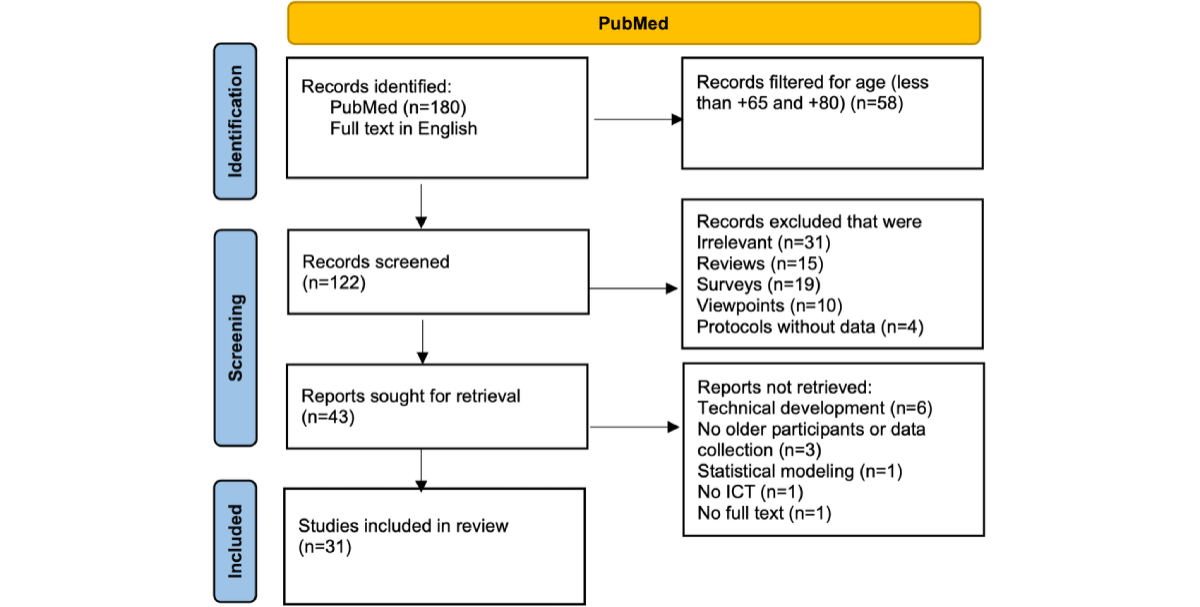
PRISMA (Preferred Reporting Item for Systematic Reviews and Meta-Analyses) flowchart to describe study selection. ICT: information and communication technology.

A PRISMA (Preferred Reporting Item for Systematic Reviews and Meta-Analyses) chart summarizes the process of study selection (Figure 1). The search (date: July 13, 2022) returned 180 articles, of which 122 were related to the age groups +65 and +80 years. We excluded reviews (n=15), surveys (n=19), viewpoints (n=10), and other irrelevant studies (ie, those that used technologies that did not have any ICT or those whose main objective was to test a functional domain in older adults using a nonassistive ICT used for data collection or stimulation presentation; n=31). We then read the remaining 43 articles and further excluded those that did not include qualitative or quantitative research methodologies. This left us with 31 articles that were analyzed qualitatively (for content, theme, and discourse).

Table 1 summarizes the reviewed literature with respect to the sampling, study design, and research objectives. See Multimedia Appendix 1 [67-97] for the descriptions of these studies.

**Table 1:** Summary of reviewed articles.

Recruited or dropout, n	Title	Framing (objective)	Study	Flexibility and playfulness	Longitudinal	Psychometrics	Inclusion or diversity
153 or 28	ICT^a^-based system to predict and prevent falls (iStoppFalls): results from an international multicenter randomized controlled trial [67]	Healthy aging (physical activity)	RCT^b^	—^c^	✓ (16 weeks)	—	CD-OA^d^; 65+ years; multinational (Germany, Spain, and Australia); 61% women
106 or 18	Use of accelerometry to measure physical activity in older adults at risk for mobility disability [68]	Healthy aging (physical activity)	RCT	Calibrated to physical ability	✓ (6 and 12 months)	Memory; health activity	CD-OA; 70-89 years; multicenter (United States); 67% women
57	A Kinect-Based Interactive System for Home-Assisted Active Aging [69]	Healthy aging (physical activity)	Quantitative	—	✓ (15 days)	—	CD-HOA^e^; 65-80 years; single center (Spain); 49% women
116	Moving real exergaming engines on the web: the webFitForAll case study in an active and healthy aging Living Lab environment [70]	Healthy aging (physical activity)	Quantitative user study	Living lab	✓ (12 weeks)	—	CD-HOA; 70+ years; 79% women
48	Use of a technology-based system to motivate older adults in performing physical activity: a feasibility study [71]	Healthy aging (physical activity)	Mixed methods (user survey + thinking aloud method including 12 HCPs^f^)	Tailored	—	—	Cognitively HOA; 65+ years; multicenter (Belgium); 58% women
249 or 48	The My Active and Healthy Aging ICT platform prevents quality of life decline in older adults: a randomized controlled study [72]	Healthy aging (sleep, nutrition, cognitive, psychosocial, physical, sleep)	RCT	Personal mobile phones	✓ (0, 6, and 12 months)	Depression risk of fall life satisfaction	CD-HOA; 60+ years; multicenter (Italy, Germany, Spain, Austria, Australia, and Japan)
30	Exergames designed for older adults: A pilot evaluation on psychosocial well-being [73]	Healthy aging (physical activity)	RCT	Gamification	✓ (6 weeks)	Self-efficacy; loneliness; life satisfaction	HOA; 65+ years; multicenter (Singapore); 70% women
16	Increasing physical activity in older adults using STARFISH, an interactive smartphone app; a pilot study [74]	Healthy aging (physical activity)	Quantitative + focus group	Gamification and socialization; personal app	✓ (6 weeks)	Step count	HOA; 65+ years; Scotland; 50% women
44 or 11	Tablet-based strength-balance training to motivate and improve adherence to exercise in independently living older people: a phase II preclinical exploratory trial [75]	Healthy aging (physical activity)	Quantitative, controlled but not fully randomized	At-home trial; gamification; personal app	✓ (12 weeks)	Technology familiarity exercise habits	CD-HOA; 65+ years; Switzerland
120 or 11	A web-based multidomain lifestyle intervention for older adults: the eMIND randomized controlled trial [76]	Healthy aging (nutrition, cognition, and exercise)	RCT	At-home intervention (personal app) and monitoring (wearable)	✓ (6 months)	Memory, depression, and chronic illness	CD-OA; memory complainers; 65+ years; France; 58% women
192 or 88	Effects of technology use on aging in place: the iZi pilots [77]	Independent living (quality of life)	Controlled trial but not randomized	Tailored to needs at home	✓ (0 and 12 months)	Self-efficacy; life satisfaction	CD-OA; 55+ years; the Netherlands
19	Matching gerontechnologies to independent-living seniors’ individual needs: development of the GTM tool [78]	Independent living	Participatory action research including caregivers	Interpreters for non-Dutch speaking participants (n=3)	—	—	CD-OA; 60+ years; the Netherlands; 58% women
9	Older individuals’ experiences during the assistive technology device service delivery process [79]	Independent living	Qualitative	—	✓ (0, delivery, and +2 months)	—	CD-OA with mobility needs; 69-91 years; Norway; 55% women
12	Older adults’ medication management in the home: how can robots help [80]?	Independent living	Mixed methods (Interview + attitudinal user-study instruments)	Living lab	—	—	CD-OA; Atlanta, Georgia; 50% women
17	“Are we ready for robots that care for us?” Attitudes and opinions of older adults toward socially assistive robots [81]	Independent living	Mixed methods (technology acceptance + focus group)	Different robot types with different andromorphic features	—	—	HOA and MCI^g^; France; 67% women
11	Acceptance of an assistive robot in older adults: a mixed-method study of human-robot interaction over a 1-month period in the living laboratory setting [82]	Independent living	Mixed methods (technology acceptance + interview)	Living lab	✓ (4 sessions)	—	HOA and MCI; France
21	Significant challenges when introducing care robots in Swedish elder care [83]	Independent living	Interviews (attitude study)	—	—	—	37-79 years; Sweden
35	Robotic services acceptance in smart environments with older adults: User Satisfaction and Acceptability Study [84]	Independent living	Quantitative user study	6 different robotic services; 3 different environments (home, outdoor, condo)	✓ (3 months)	Memory activity	HOA; 65+ years; Italy; 63% women
2797 or 344	Healthy aging through internet counseling in the elderly (HATICE): a multinational, randomized controlled trial [85]	Healthy aging (cardiovascular health)	RCT	—	✓ (18 months)	Memory, depression, and chronic illness	65+ years; the Netherlands, Finland, and France; 47% women; 97% White
Same as above	Factors predicting engagement of older adults with a coach-supported eHealth intervention promoting lifestyle change and associations between engagement and changes in cardiovascular and dementia risk: secondary analysis of an 18-month multinational randomized controlled trial [86]	Same as above	Quantitative, engagement study	—	—	—	65+ years
14	Triggering postural movements with virtual reality technology in healthy young and older adults: A cross-sectional validation study for early dementia screening [87]	Risk management	Quantitative (gait analysis, HOA vs HYA^h^ [n=15])	—	—	Memory	HOA vs YA^i^; 65+ vs 23+ years; Switzerland; 57% women
33	Machine-learning approach to predict on-road driving ability in healthy older people [88]	Risk management	Quantitative	—	—	Memory, vision, attention, and depression	HOA; 65+ years; Japan; 93% men
45	Gerontechnology: providing a helping hand when caring for cognitively impaired older adults-intermediate results from a controlled study on the satisfaction and acceptance of informal caregivers [89]	Risk management and independent living	RCT	Home installations	✓ (3 time points; 15 months)	Memory	CD-OA; with MCI; 60+ years; Greece, Denmark, Finland, and Ireland
12	Multi-stakeholder perspectives on information communication technology training for older adults: implications for teaching and learning [90]	Independent living	Interviews and multistakeholder focus groups (with 14 care providers)	Individualized home-training program	✓ (0 and 24 months)	—	CD-OA; 70+ years; New Hampshire, United States; 91% women
184	Aging well in the digital age: technology in processes of selective optimization with compensation [91]	Well-being	Focus groups	—	—	—	65+ years; Canada, Colombia, Israel, Italy, Peru, Romania, and Spain; 100% women
22	Pilots for healthy and active aging (PHArA-ON) project: definition of new technological solutions for older people in Italian pilot sites based on elicited user needs [92]	Healthy aging	Mixed methods (user study + interviews), including formal and informal caregivers too (n=39)	—	—	Memory	Frail and MCI OA; 65+ years; 2 sites (Italy); 59% women
13	A qualitative study toward technologies for active and healthy aging: A thematic analysis of perspectives among primary, secondary, and tertiary end users [93]	Home care	Thinking aloud and focus group with caregivers	—	—	—	HOA; 65+ years; Italy and Romania
6	What it takes to successfully implement technology for aging in place: focus groups with stakeholders [94]	Home care	Focus group (other stakeholders; n=23)	—	—	—	HOA; 63+ years; the Netherlands; 50% women
21 or 9	Evaluation of 1-y in-home monitoring technology by home-dwelling older adults, family caregivers, and nurses [95]	Home care	Mixed methods (user study + interviews, including stakeholders)	At-home trial	✓ (12 months)	—	CD-OA; 70+ years; Switzerland; 47% women
30	“What? That’s for Old People, that.” Home adaptations, aging and stigmatization: A qualitative Inquiry [96]	Home care	Focus group (+ professionals) + observational from wearable cameras	—	✓ (6 months)	—	65+years ; 2 sites (England); 57% women
11	User-centered development of a web Platform Supporting Community-based health care organizations for older persons in need of support: Qualitative Focus Group Study [97]	Home care	Focus group (+ caregivers + professionals)	—	—	—	67+ years; Switzerland and Slovenia

^a^ICT: information and communication technology.

^b^RCT: randomized controlled trial.

^c^The study did not use this method.

^d^CD-OA: community-dwelling older adults.

^e^CD-HOA: community-dwelling healthy older adults.

^f^HCP: health care professional.

^g^MCI: mild cognitive impairment.

^h^HYA: healthy young adults.

^i^YA: young adults.

#### What Types of Research Methods and Recruitment Strategies Are Used in This Line of Research?

Table 2 provides an overview of the scope of the methods, objectives, and outcomes in the reviewed literature.

**Table 2:** Summary of content and thematic analyses (N=31).

Source and content		Counts, n (%)
**Background and rationale**		
	Growing cost of caring for age-related deficits	12 (39)
	Age-related disabilities	14 (45)
	Desire for independent living	12 (39)
	Prevention toward healthy aging	10 (32)
**Study design**		
	Focus groups	9 (29)
	Mixed methods	10 (32)
	Quantitative trials (including RCTs^a^)	10 (32)
	Observational studies	2 (6)
**Methods of recruitment**		
	Not described	18 (58)
	Random sampling	1 (3)
	Convenient (selective and targeted)	12 (39)
	Multistakeholder	8 (26)
	Multinational	4 (13)
**Results and outcomes**		
	Impact on physical fitness	10 (32)
	Acceptance of home adaptation	9 (29)
	A-ICTs^b^ for early detection of age-risks	5 (16)
	Future of A-ICTs	8 (26)

^a^RCT: randomized controlled trial.

^b^A-ICT: assistive information and communication technologies

#### Classifications of Study Characteristics and Sampling

The studies reviewed included qualitative focus groups (9/31, 29%), mixed methods (10/31, 32%), quantitative interventions (10/31, 32%), and 2 observational and 1 participatory studies. Furthermore, 4 studies were longitudinal and 2 were multinational.

Recruitment procedures were not described in all articles; however, among those that did (n=13), only 1 study involved a random sampling strategy from a general population pool. The remaining (12/31, 39%) used selective and targeted methods of recruitment by reaching out to a specific group of older adults whose needs were a priori known to clinical partners or organizations that care for older adults.

Participant characteristics were often biased by the inclusion criteria, which either included those older adults without cognitive or physical disabilities or those with such conditions. Only 14 studies specified the health conditions of the participants in the study sample. Eight studies recruited only healthy older adults; 3 studies recruited participants with cognitive impairment, 2 of which also had healthy older adults as controls; and 1 included older adults with disabilities.

Gender was also a biasing factor, with women being overrepresented in the study samples among the 10 studies that reported the gender ratio. In addition to limitations in the representativeness of the samples, further biases were noted in retention and attrition, which will be addressed in the Discussion section.

### Which Discourses and Objectives Drive the Study Rationales and Objectives?

#### Classification of Rationales

Most of the articles reviewed framed the research in the context of the burdens of aging (cognitive deficit, physical disability, dependence, frailty, and isolation). Among the articles included in this review, 39% (12/31) of articles began by describing concerns about the rising costs of the growing older population; 45% (14/31) began by discussing age-related disabilities such as dementia and frailty; 39% (12/31) focused on desire for independent living or aging in place; and 29% (10/31) framed the study in the context of preventive measures to promote successful aging.

#### Classification of Research Objectives and Specific Aims

In terms of the objectives of research, 4 general categories emerged: studies that aimed to assess A-ICT–based interventions to promote healthy aging by physical activity (n=10); studies that evaluated the acceptance and feasibility of introducing A-ICTs such as robots and remote health monitoring systems to promote independent living (n=9); studies that validated the ability of A-ICTs to detect age-related risks caused by physical or mental deficits (n=5), and several multistakeholder focus groups or ethnographic studies about the future of A-ICTs in health care for older adults (n=8). See Multimedia Appendix 1 [67-97] for the descriptions of these studies.

### Are the Study Elements Communicated in Compliance With the Non-Ageist Guidelines for Research?

Using the recommendations of the Task Force to Develop Non-Ageist Guidelines for Research [28] as a reference, we examined the extent to which the reviewed studies were compliant with the following questions:

#### Did the Study Treat Age in and of Itself as an Appropriate Explanatory Variable?

This was not the case in most studies reviewed here. Only 2 studies referred to age as an explanatory variable to validate automated measurement systems with the assumption that older adults have functional deficits compared with young adults [78,87].

#### Were the Instruments Used in Research Biasing and Did They Equate Age With Decay, Deficit, and Death?

We found that some of the quantitative studies framed within the discourse of successful aging included psychometric instruments specific to deficits of older populations, such as assessing cognitive deficits (eg, the Montreal Cognitive Assessment test and the Mini Mental State Examination) [76,84-89,92,95], geriatric depression [72,76,85,86,88], chronic illness and disability [68,75,76,85,87], risk of falls [67,68,72,75], and other instruments that measured presumed age-related variations such as loneliness [73], self-efficacy, and self-reliance [67,73,77] quality of life and life satisfaction [67,72,73,77].

#### Did the Examiners Possess a Perspective on the Life Stage of the Participants in Their Studies?

Given that sampling in the reviewed reports was not entirely random, it is plausible to assume that examiners possessed some perspective on the life stages of older adults. Among the quantitative studies in this review, awareness of life stage was operationalized in terms of the quantitative assessment of aspects of individual life being affected by age (as described above). Among qualitative and design-related studies in this review, attention was focused on capturing the existing and emerging needs and attitudes of older adults. This manifested in terms of multistakeholder study designs [90,94], evaluating factors such as digital literacy [76,90,91,93], history of ICT use [90,91,96], technology acceptance [69-71,78,81-83,89-91,93,97], and tailoring the interventions to individual needs [75,77,78,93,94,96], which implied that older adults were less likely to be aware of new technological developments.

#### Was the Language Used to Describe the Results Value-Laden, Especially When the Findings Gain Attention From Media or Policy Makers?

Some statements in the conclusion may be construed as value-laden. For example, referring to the aging population under study as “elderly” is no longer culturally accepted. The word “elderly” was in the abstract, keyword, and conclusion of some studies [68-70,73,85,86,89,94]. Referring to the population as “older people” objectifies and segregates them. The following examples, all from studies involving technology development, demonstrate this objective distancing, which makes the aging body part of the machinery that was invented and tested:

The adoption of assistive technology devices for physical intervention tends to motivate and retain older people who exercise for longer periods of time

75

Our findings led to some suggestions for robot designers to make assistive robots more attractive and acceptable to older people

82

Our model successfully dissociated unsafe drivers from safe drivers with an accuracy of 90.9% (sensitivity of 75.0% and specificity of 100.0%), suggesting that aging, decline in attentional and visuoconstructional functions, and reduction in functional visual acuity are strongly associated with a high risk of unsafe driving among healthy older people

88

Another possibility for value-laden conclusions is the emphasis on deficits and generalizing it to a population. These deficits may be related to individual conditions, such as dementia or learning disabilities, which are the most feared stigmatizing notions. Examples of such statements include:

To establish the new assessment system as a diagnosis tool for dementia in the future, we will improve the research design as discussed above and conduct additional measurements with people suffering from dementia to understand more specific and relevant parameters to diagnosis

87

Different types of technological solutions are needed, depending on individual personal factors. Furthermore, it is important that the system works with minimal interaction and with automated operations because of limited learning abilities among the users or because they have very little experience with the new technologies

89

### Is Age as a Selection Criterion Informative?

#### Overview

The majority of studies in this review expressed a general concern for the impending costs of aging and recruited based on age criteria. However, age was not used as a predictive or explanatory variable. Only 1 study conducted random sampling. The rest performed convenience or selective sampling within existing pools of individuals who participated in other geriatric care programs. Even within these pools, the rates of participation and attrition varied. Studies that specifically focused on medical conditions such as frailty and dementia were purposively recruited too.

This observation reinforces our concern that participation in age-related A-ICTs may be biased by the research questions and methodology. We identified the following factors to be more important than age.

#### Context of Research

The objectives of promoting healthy or successful aging, cost reduction, independent living, and risk reduction were the primary motivations for the studies reviewed here. However, the findings of most studies are not strongly conclusive, especially those of randomized controlled trials (RCTs). The variability in the results was mainly related to acceptance and adherence, but not age.

For example, in a large-scale clinical trial of ICT-coached cardiovascular risk management, the largest effects were observed in those who had the ability to access and engage with technology [85]. A similar RCT investigating the impact of tablet-based physical training reported that attrition was related to individuals’ appraisal of what they expected from the app as well as to the motivational factors related to social interactions [75]. In other words, *the context* in which an ICT-based intervention took place was more effective in promoting adherence than ICT functionality.

The circumstances and contexts of individual experiences seemed to shift the statistical results. For example, an RCT of an ICT-based exercise program tried for 8 weeks by home-dwelling older adults reported that group differences were not due to improvements in the intervention group but due to a decline in the control group [67]. A subgroup analysis revealed that the benefits of the intervention in the high-adherence group were more pronounced than in others, thus calling for future studies to explain individual differences in adherence to the intervention. Another RCT involving a web-based multidomain lifestyle intervention did not find any significant functional improvements in participants and observed that, despite accepting the conditions of the trial, adherence (especially with physical exercises) requirements were not met. They speculated that this was linked to motivational factors not measured in the trial [76].

#### Agency and Support

Insights from the multistakeholder focus group study of *Welfare Technologies* that offer assistive robots to the Swedish older population [83] point to another important source of bias: individual perceptions of agency and control. This study found that not age but the perceived absence of ethical and governance frameworks, as well as lack of collaboration and health spending, were impediments to acceptance, access, and successful adoption of the proposed technologies.

In fact, tailoring interventions to individual needs was found to be important in a longitudinal RCT that introduced a wearable ActiGraph to monitor the physical activity of prefrail older adults [68]. In another RCT of an ICT-based frailty prevention study, physical fitness in the intervention group did not increase, but quality of life in the control group declined. The study did not have any qualitative data to offer any explanation of the change in the control group but called for future examination of the neurobiological mechanisms of this effect [72].

A multinational acceptance study (local communities in Greece, Finland, United Kingdom, and Denmark) of an “intelligent” telecare system for independent living and self-care of older adults with mild cognitive impairment explained the challenges of recruitment (achieving <25% of the targeted sample size) [89]. Interestingly, this study observed significant regional differences in service use, as well as regional and personal variations in service appreciation. It was noted that learning to operate a new automated system for those with cognitive impairment is impractical, and if such interventions are offered, they need to be offered in a personalized manner to individuals who can benefit from them.

This study is laudable for providing a very detailed picture of the challenge of recruiting representative samples within the predefined clinical criteria and explaining deviations from initial study design and recruitment caused by the reality of heterogeneity not only among the needs of individuals with dementia, but also heterogeneity of the technology literacy of caregivers (family or nurses) and the health care systems within which they receive care [89]. In conclusion of their report, the authors have noted the following:

We can confirm that it is of high importance that the primary user and caregivers to be motivated toward use of aiding technologies in their homes. For the acceptance of the services by the elderly, a key role plays their family caregiver and the process is much faster and easier if the caregivers have previous experience with technology.

#### Self-exclusion and Perceived Stigma

A user-centered phenomenological study of older adults’ experience during assistive technology device (ATD) delivery [79] revealed that the framing of the study in the context of age-related deficits was in and of itself a biasing factor in recruitment:

The recruitment personnel reported that the reasons for declining participation included a lack of comfort discussing disabilities and the binding commitment to the project necessitated by the length of the study.

In this study, the perception of self-deficits and fear of ageist attitudes contributed to reluctance to participate:

For some participants, contacting the occupational therapist because they needed additional help was considered to mean that they would be perceived as rude, ungrateful, and subject to negative consequences. [...] One of the participants said that a previous comment about assistance that she had received resulted in retribution from the health care professionals. [...] Because of her fear of jeopardizing her relationship with the health care professionals on whom she depended, the participant simply put up with the situation when she received an ATD [assistive technology device] that she did not know how to use.

This study concluded that satisfaction with assistive technology is not easily measurable and that “there are several complicating reasons for older individuals not to acknowledge unsatisfactory experiences in the service delivery process...related to expectations, disappointments, fear, and abandonment but also hope, mastery, and resourceful and dynamic self-management of care.*”*

Another qualitative study across 2 sites (in the United Kingdom) with 30 older adults and 39 nonfamily caregivers undertook purposive sampling to capture the diversity in minor or major home adaptation and its funding source: age range (65-74, 75-85, and >85 years), ethnicity, gender, household composition, house type, and tenure. This study noted that stigmatizing notion of aging (equating it with vulnerability and disability) was an impediment to learning about and seeking technologies to adapt homes to the needs of older adults. Participants in their study showed awareness of the “ageist” attitudes, and some expressed a fear that to use assistive technologies (as neutral as a staircase railing) would make them “appear old” or signal frailty and disability, which would lead to stigmatization [96].

Refusal to participate in the study of a home-monitoring installation study by 54 of 127 eligible candidates, and the completion rate of 12 of 21 in those who enrolled in a year-long trial point to other sources of bias [95]. Refusal to participate may also be implicit, for example, by not receiving a response from more than 39,000 of the 45,466 invited individuals (with 696 explicit refusals) [85]. Therefore, it is not surprising that positive results are reported within a highly motivated sample who showed interest in adopting home monitoring strategies.

### What Strategies Were Used to Avoid Age Stereotypes?

#### Participatory Research

Notwithstanding the limitations in sampling discussed above, several studies within this review have strived to conduct participatory and inclusive research, especially regarding the future of A-ICTs. Strategies used to accomplish this included conducting focus groups involving different stakeholders [92-98].

#### Need-Based Recruitment

In recognition of the fact that age does not capture the heterogeneity of needs within older populations, several studies in this review took a need-based approach to studying the affordances of A-ICTs [77,78,92,94].

#### International Sampling

Conducting research across different national or regional jurisdictions was an important strategy to demonstrate not only the diversity of older adults’ needs but also the differences in institutional and socioeconomic contexts in which they lived [89,91,93,96].

#### Longitudinal Designs

Longitudinal follow-up in several of the studies reviewed here ranged from 6 to 12 weeks to 12 to 18 months. The longer the duration of the study, the greater the possibility of examining the evolving relationship between the users, caregivers (family or professional, such as nurses or social workers), and the technologies presented [75-77,79,82,89,95,98].

#### Providing Choice of Options

Presenting different real [84] or hypothetical [94] options to users or evaluating A-ICTs within simulated environments such as living laboratories [81,82,88] added flexibility to the research frameworks. The more options and the greater the opportunities to “play,” the greater the chances of retaining participants and recording positive experiences.

#### Personalizing Interventions

Tailoring the intervention individual needs [75,77,78,93,94,96], “technology matching” [78], or calibrating the intervention to the cognitive or physical ability of the participants [68,71] were important in addressing the heterogeneity within the sample.

#### Creating a Safe Space to Receive Feedback From Participants

Adding interviews within a safe space encouraging participant’s candid and critical views also led to the discovery of factors that could bias the research, such as motivations [74], tensions such as fear of stigmatization [79,83], or fear of losing human touch [80], thus extending the findings beyond age-related explanatory variables, such as physical and cognitive ability, and technology use.

## Discussion

### Summary of Findings

To the best of our knowledge, this is the first systematic mapping review of the literature at the intersection between successful aging, A-ICT, and health care. We performed content, thematic, and discourse analyses in 31 selected research studies, trying to answer the following questions: What types of research methods and recruitment strategies are used in this line of research? Which discourses and objectives drive the rationales and objectives of the study? The study elements were communicated in compliance with the Non-Ageist Guidelines for Research [28]. Is age an informative selection criterion? Which research strategies should be used to avoid age-related stereotypes?

Our search strategy was successful in retrieving diverse studies. This review included several categories of research studies, using both qualitative and quantitative research methods and diverse study designs (longitudinal, multinational, RCT, focus group, phenomenological, and user experience). As such, it provides a broad overview of the methodological approaches taken to address the question of the affordances of A-ICTs for successful aging.

Overall, discourses on aging as a problem were prevalent. The rationales classified from the thematic analysis of the Introduction section were primarily framed in the discourse of age-related deficits, rising costs of care for a growing aging population, quality of independent living, and safety. Thematic analysis also resulted in 4 categories of objectives and expected outcomes that contribute to healthy aging: promotion of physical activity, facilitation of independent living (primarily by the introduction of robots), monitoring of age-related deficits, and envisioning the future of A-ICTs in geriatric care. Interestingly, however, while research was often framed with the aim of assisting the general population of older adults, both the recruitment and the findings of most studies indicated that only *some* study participants could benefit from the proposed intervention.

We observed disciplinary differences in attentiveness to nonageist research guidelines. Participatory, multistakeholder, and multinational studies that undertook qualitative research were more attentive to the heterogeneity in attrition, adherence, and acceptance related to the context of the research, individual needs, agency, and availability of support. In contrast, quantitative or RCTs characterized sample heterogeneity in terms of differences in health states and mental or physical abilities. In neither research category was the age of a predictive or explanatory value. However, in this review, there was evidence that fear of ageism, or self-ageism, biased recruitment.

### Implications for Future Research

Mapping reviews provide an opportunity to examine the bigger picture of a research area to pinpoint specific gaps in knowledge that might require more complete systematic reviews or propose guidelines to be considered in future practices [99]. In Table 3, we summarize some of the topics that can benefit from systematic or scoping reviews.

This review corroborates previous research that recruiting older adults into research is challenging [30,100] and that trust-building and cultivating community-based communications are critical factors in keeping participants interested in the study [100]. This review also underlines the fact that the successful adoption of A-ICTs requires resources for human support to help prospective users overcome technostress and the steep curves of learning and mastery [46,57-59,98]. Thematic analysis of research rationales and objectives in this mapping review illustrated the risk of double-binding arising from miscommunication or misunderstanding of research objectives. Further discourse analysis of research discussions showed the strength of interdisciplinary and flexible research frameworks that mitigate the biases arising from the double-bind research age. We discuss the information that we have synthesized as opportunities for improving research design and contextualize it with reference to the previous body of knowledge.

**Table 3:** Research strategies that can mitigate ageism.

Suggestions	Benefits	Examples
Interdisciplinary approached and mixed methodologies for data collection	Allow to gather both qualitative and quantitative data to explain variations in both functional domains that can be objectively measured (eg, sensory, cognitive, and physical abilities), as well as perceptual and attitudinal factors that predict acceptance, adherence, and engagement.	[70,71,74,80-82,92,95]
Personalized, recursive, and longitudinal study designs	Introducing A-ICTs^a^ into one’s lifestyle involves a process of negotiation among designers, caregivers, and users. The dynamics of relationships between these parties change with personal factors, and with time as they become familiar, evaluate, and fit them to their needs.	[68,71,72,75-82,89,91,95,96]
Playful designs	Conducting research in simulated environments and providing users the ability to approach A-ICTs in a playful manner helps mitigate the possibility of feeling pressurized to perform or successfully adopt.	[70,73-75,80,82]
Framing research in needs rather than age	Avoid generalizing titles, especially if research is tied to specific needs such as frailty or dementia. Designing solutions that are universal and inclusive for needs will avoid creating age-related stigma.	—^b^

^a^A-ICT: assistive information and communication technology.

^b^Not available.

#### The Necessity for Interdisciplinary and Mixed Methods Approaches

Clearly, the question of how to create an ICT to assist the aging population poses one of the most complex questions at the intersection of many fields: medicine, interaction design and communications, health psychology, sociology, and engineering. This inherent interdisciplinarity in and of itself presents challenges in developing an account of complex systems dynamics [101].

This review indicates that the simpler the targeted behavior (eg, increasing physical activity), the more likely it was that the study reported satisfactory outcome [67-70,74,75]. However, the introduction of other technologies such as assistive robots or the introduction of technologies for independent living resulted in ambiguity in the interpretation of findings. The inconsistencies were caused by individual differences in attitudes, abilities, and expectations tied to prior knowledge, experience, and care systems [73,80-86,90,91,94,95,96]. Physical activity is beneficial for health and is widely accepted irrespective of age. That robots and machines may replace humans is received with ambivalence and therefore more variations in acceptance are expected.

The inclusion of both quantitative RCTs and qualitative observational or focus group studies in this review uncovered an interesting disciplinary gap to bridge. For example, quantitative studies on the benefits of interventions for healthy aging often control for variations in emotional, physical, and psychological factors. However, these studies rarely accounted for participant’s views, cultural backgrounds, lifestyles, and socioeconomic resources available to them. Conversely, design-oriented and participatory studies that focused on contextual and socioeconomic factors that shape attitudes rarely controlled for cognitive, physical, and affective variations that shape one’s particular needs.

Interestingly, a discourse analysis of the language used to describe research objectives and conclusions revealed that overly reductionist studies are more likely to deviate from the recommendations of the Non-Ageist Guidelines for Research. Studies designed with qualitative and participatory components were generally more careful in discussing the needs of older adults, primarily focusing on the desire to live independently in later years of life. In contrast, more technical articles used a more “objective,” and potentially objectifying tone in describing the research rationale and results. For example, in a study that utilized machine-learning approaches to identify risky drivers, the authors included age as a clinical variable and concluded that age-related decline was predictive of unsafe driving [88]. In another study, to validate a virtual reality–based diagnostic tool for the early detection of dementia, researchers included 2 groups of young and healthy older adults on the presumption that the system would be sensitive to detecting age-related deficits in the latter group [87].

The concept of assistive ICTs for health care for older adults is new and still in the design phase, and tensions in personalizing or generalizing care are pervasive [94]. Therefore, interdisciplinary and cocreation approaches may achieve a higher research impact and accelerate the development of more effective solutions [81].

#### The Necessity of Conducting Flexible Research

RCTs are preferred by scientists seeking evidence for valid, reliable, reproducible, and effective interventions. Given this wide acceptance, it is perhaps surprising that even in purely medical interventions, contemporary researchers have questioned the validity of making inferences from RCTs [102,103]. As Fink and Keyes [104] have shown, ignoring complexity, especially in public health science, can lead to erroneous inferences and shift the ground on which scientific inquiries claim truth: reproducibility. This review included 6 RCTs, 4 of which focused on physical activity [67,68,72,75] and 2 on general behavioral coaching to promote a healthy lifestyle [76,85]. Overall, recruitment, retention, and adherence of participants presented challenges. For decades, medical anthropologists and other social scientists have pointed out the limitations of RCTs in studying interventions that depend on culture and context, especially interventions that target the psychosocial well-being of populations [105]. The fact that a larger proportion of studies in this intersectional study were non-RCTs points to the complexity of the problem and the fact that reductionist methodologies are not sufficient.

In RCTs, it is difficult to account for all the personal factors that shape one’s behavior, let alone their relationship with new technology. Among older adults, Birkland [106] has synthesized data from extensive interviews to categorize users under labels Enthusiasts, Socializers, Practicalists, Traditionalists and Guardians—with the latter groups being the most likely to find new technologies stressful [107]. These factors influence attrition. For example, in a mixed methods study of an ICT-based behavioral intervention to increase physical activity [74], those who completed a 6-week intervention were highly interested in the intervention. The study reported that the mean daily step count increased from 9443 (SD 3952) steps before the intervention to 10,773 (SD 2659) steps after the intervention, with a mean increase of 14%. It is important to note that these effects were not statistically significant. In this study, one participant decreased their step count from 15,611 to 14,772 (already higher than the commonly recommended 10,000 daily steps). These numbers, as well as comments recorded from the participants, indicate that participants whose data acquisition was successful (8/16, due to technical failure) were highly motivated to engage with this activity, a reality that is not considered in statistical power calculations to recommend the intervention for RCT.

Similar to clinical researchers, who seek objective evidence to support the universality of the effectiveness of their intervention, designers strive to satisfy the universal design principles of *equity* (useful strategies for a wide range of users independent of age), *flexibility* (accommodating a range of preferences and methods), *simplicity* (independence from literacy, skills, or language), *perceptibility* (clearly communicating their purpose and use case), *failure-safety* (minimizing risks of error), and *accessibility* [108]. For this reason, holistic and participatory research practices help refine applications through recursive evaluations and improvements [92,109,110].

#### The Necessity of Conducting Recursive Research

In the Introduction, we pointed out that double-binds may be caused by miscommunication, which can be corrected and negotiated over time. Considering the dynamics of change in behavior requires flexibility in methodologies that can capture patterns of change. For instance, longitudinal studies within this review illustrate that technology acceptance is a dynamic process that begins with recruitment (ie, who chooses and refuses to participate) and evolves through experimentation and trial, as study participants establish relationships with the researchers. Indeed, in a study where the acceptance of robots for medication delivery was investigated [80], despite growing acceptable over time, ultimately, the users did not find such technology to be a suitable replacement for human care. Monitoring attrition over time, as well as monitoring the relationship dynamics during research, provides insight into the context of how needs, perspectives, and levels of engagement change [72,75-77,79,81,82,89,91,95,96].

A phenomenological study of older adults’ experiences during assistive technology service delivery further underlines the importance of taking a recursive approach to research [79]. Before receiving the assistive technologies that the participants had applied for, they were optimistic and hopeful that the technology “would make life easier and would enable them to perform their desired activities. The participants were confident they would be able to manage using the ATD*.”* However, after receiving the devices “their encounter with their ATD and the person who delivered it either confirmed the participants’ positive expectations (if they could manage to use it correctly) or surprised them when their expectations that the ATD would ease their everyday life were not met.*”* Further examination of explanatory variables (such as the self-described personality of users who called themselves assertive) showed between-individual differences in the process of adoption (taking charge and seeking help or putting up and being dissatisfied).

Conducting research over time allows researchers to examine how individuals cope with the technostress of new experiences. Consider learning as an example. Several studies in this review found that, in addition to acceptance, the desire or ability to overcome the challenge of learning can themselves become sources of dissatisfaction and potential stress [78,82,89,90,93,97]. Independent of age, a large proportion of eHealth solutions suffer from high rates of dropout, discontinuation of use, or nonadoption [111]. Therefore, it is important to develop tools to match gerontechnologies to individual needs is important [78]. Incorporating Lazarus’ transactional model for stress and coping offers an empirical framework for evaluating the influence of personal factors in the appraisal, adoption, or rejection of new interventions [112,113].

#### The Necessity of Conducting Playful Research

Capturing individual preferences in real-life situations is important. A mixed methods study involving the introduction of 6 robotic services in a realistic living environment [84] provides an example of how playful and recursive approaches may be informative. In this study, participants were given the freedom to become familiar with the robots before starting the experimental session to feel more confident in testing them. Interestingly, this study found that older adults enjoyed the anthropomorphic design of the robot (with a moving blinking head), which is conducive to interaction. According to the authors, *“*Only two users did not get pleasure in testing the Robot-Era system because they claimed to see the robotics system as an appliance that is used for its usefulness and not for pleasure.” It is plausible to suggest that the playful nature of the task of evaluating various types of robots, without pressure to adapt them to their real lives, was conducive to better interaction with the systems [84].

Besides providing an opportunity to learn from simulation, adding playfulness to the research itself mitigates the discomfort that arises from perceived social evaluative threats in researcher-participant relationships [67,70,83]. When technology is introduced through a serious health care context, the risk of self-censoring owing to the fear of losing potential privileges increases [79]. Conversely, playfulness can help free expressions of actual needs and attitudes. For example, introducing assistive robots with the specific aim of assisting individuals with mild cognitive impairment resulted in rejection of the notion that such services could replace humans for caregiving, whereas those with mild cognitive impairment seemed to enjoy the playful features of the technology in proving distraction while also being concerned about the image portrayed by using a machine for companionship [81].

Adopting a playful framework mitigates performance stress, increases enjoyment, and provides an opportunity to make observations about choice, socialization, mastery, and self-efficacy to help envision more inclusive and user-centric interventions.

### Limitations and Future Directions

This review has several limitations that necessitate further research.

First, the research questions that motivated this study arise from a subjective perspective that is informed by the authors’ experiences while conducting quantitative, qualitative, and community-based research on older adults’ use of technology in general, and its implications in their health care in particular. The search criteria were tied to these questions.

Second, mapping reviews do not aim to investigate the quality of the reviewed research; rather, they detect and categorize themes that emerge from those studies to sensitize researchers about future possibilities. The conclusions of this review are not tied to specific objectives and cannot be generalized. Further systematic reviews are required to confirm these conclusions.

Third, our search was limited to 1 database (PubMed), and the search logic was narrow. This decision was made to focus on the intersection between the notions of “successful ageing” and “Assistive ICTs” with “older adult” as key terms. As such, the studies collected through this method do not represent the entire field of aging with technology. We acknowledge that additional information or different conclusions could have been obtained if other databases were searched.

Finally, it should be noted that our search strategy returned studies that were mostly conducted in Europe. As such, they do not include trends that may exist in other international contexts. Whether this continental bias is related to our research questions or to the research agenda of different countries needs to be further investigated.

### Conclusions

Our systematic mapping review illustrates that conducting or reporting research under a generalizing assumption of 65+ years of age is neither practical nor informative. Table 3 summarizes the research strategies that complement existing guidelines and mitigate the risks of ageism. Synthesizing a framework based on the collective strength of all studies reviewed here, to conduct research in a flexible and longitudinal framework that is attentive to changes in personal appraisal and approach to the questions of A-ICTs and successful aging, is critical. Mixed methods research, which documents variations in physical, psychological, and socioeconomic contexts, might address the current state of inconclusiveness regarding strategies and interventions that can be effective. As several studies within this review illustrate, one advantage of designing problem-focused, need-based, and person-centered research is that it expands the possibilities of how best to serve those who seek technological solutions to improve the quality of their lives.

Adopting an eco-social framework that looks at individual needs and coping styles reveal more specific dimensions of individual variation and contexts that may influence the uptake and response to A-ICTs. For clinical and technological researchers to collaborate in participatory research is an important first step.

## Appendix

Multimedia Appendix 1 Description of studies in the review.

## Abbreviations

A-ICT: assistive information and communication technologies
ATD: assistive technology device
ICT: information and communication technology
MCI: mild cognitive impairment
PRISMA: Preferred Reporting Item for Systematic Reviews and Meta-Analyses
RCT: randomized controlled trial
