# Supplementary material for: Double-Bind of Recruitment of Older Adults Into Studies of Successful Aging via Assistive Information and Communication Technologies: Mapping Review
Source: JMIR Aging. 2022 Dec 23;5(4):e43564. doi: 10.2196/43564 (PMC9823588; doi:10.2196/43564)
Supplement: Multimedia Appendix 1 [file aging_v5i4e43564_app1.docx]

**Supplemental Material 1: Description of Reviewed Studies**

# Promoting Healthy Ageing by Using Technology for Physical Activity

The importance of physical activity for healthy ageing and reducing the risks of frailty prefaced studies of A-ICT based interventions involving monitoring or motivating exercise (n= 10).

A randomized controlled trial of an ICT-based fall prevention program tested during 16-weeks among 78 (15 drop-outs) older adults [67]; an accelerometry trial in two groups of physical activity (n=54) versus "successful ageing" group (n=52) showing accurate quantification and differentiation of meaningful activity between groups [68]. Qualitative studies of a Kinect (TM)-based exercise monitoring system in 57 participants [69], and an integrated exergaming system in a living lab, tested quantitatively in 116 older adults and qualitatively in 12 were also reported as successful [70]. Another mixed methods feasibility study of a neurological rehab ICT for physical training tested in older adults visiting a day-care centre (n=32) or living in long term care facilities (n=16), and 12 healthcare professionals showed that to personalize the system and offer a person-centred training was a critical factor in engagement with the promising system [71].

In several studies, the interventions failed to achieve improvements in physical activity for which they were designed:

A multi-national study dedicated to developing ICTs for the promotion of active and healthy ageing, included 201/636 screened pre-frail older adults from European and non-European countries in a one year trial, with a relatively high completion rate (80%) but found that interventions did not improve physical, cognitive social or sleep functions compared to the control group, although the study showed a significant difference between the two groups in terms of lowered quality of life in the control versus unchanged scores in the intervention group [72]. A quantitative pilot study of 5 exergames specifically designed for older adults aimed to show that compared to traditional exercise, these interventions will be better improving psychosocial well-being of their users (measured from self-efficacy, loneliness, and life satisfaction). While enjoyment of playing digitally was marginally improved, the impact of interventions on all other psychometric measures was non-significant [73]. Another smartphone app for behavioral change tested in older adults (n=16) showed high acceptance and moderate (although not significant) improvements in step-count of 8 participants [74]. A preclinical 12-weeks trial with a tablet-based strength training in older adults randomized to individual users (n=14 ), social users (n=13) and controls (n=17) and found that although no improvements were measurable in physical strength domains, the intervention was acceptable and its social features served as an effective motivational factor [75]. Similarly, in a longitudinal RCT in France, evaluating the impact of web-based multidomain lifestyle training in older adults (n=54) versus control group (who did not access the training site), researchers found the system to be acceptable by users, but failed to measure quantifiable improvements in functional domains (cognitive or physical) targeted by the training [76].

# Promoting Independent Living by Home Adaptations

Studies involving the introduction of technologies for increasing the quality of ageing in place were observational and qualitative and often generated more research questions emphasizing the need for taking a user-centred and needs-based approach. A large scale, study in The Hague (The Netherlands) demonstrated that through the process of technology-matching to individual needs, they succeeded in encouraging the adoption of technologies in a targeted older population in the city. This contributed to an improved sense of physical well-being among those who adopted the technology (n=115 from 294 who were offered to participate) versus controls (n=322) in a 12 month pilot study [77]. A related dual-phase participatory action research project with stakeholders concluded that the needs of older adults are diverse, and that existing technologies are limited in their ability to address personal needs and circumstance and concluded that the process of *Gerontechnology Matching* was a critical step towards development and adoption of assistive technologies [78]. Similarly, another needs-oriented study in Norway reported an evolving change of attitudes towards assistive technologies among those who had indicated a need and applied for them, resulting in a "unanimous attitude of hope and positive expectations" that according to authors contrasted the generally negative perceptions among older adults about technology [79].

Assistive robots were prevalent as possible home-care solutions to improve independent living. These studies were often conducted by designers and involved acceptance studies. A mixed methods evaluation of the acceptance of an assistant medicine dispensing robot introduced in a home-like living laboratory among 12 racially diverse older adults in Georgia showed that participants were interested in having a robot remind them to take the medication, but were not convinced that the robot could be persuasive or able to help them know which medication to take and preferred personalized human assistance to robots [80]. Similarly, another acceptance study in 25 older adults evaluating assistive social robots for persons with mild cognitive impairment showed ambivalence in the desire to adopt such potentially useful technologies, partly due to fearing the stigmatizing image of the solution [81]. While the healthy older adults thought that the robots might be useful to them in the future (when and if they need them), those with a diagnosed cognitive impairment feared that robots would replace humans and impact their autonomy negatively [81]. This ambivalence was corroborated by the findings of another one-month intervention in six older adults with mild cognitive impairment and five cognitively healthy controls of the same age range. The study indicated that both groups were not interested in using the robot and had a negative attitude towards adoption of the robot, triggered by fearing stigmatization due to association of use of robot with disability [82]. A multistakeholder focus group study of barriers to adoption of *Welfare Technologies* (assistive robots provided to older adults) revealed that besides acceptance and attitudes, the absence of legal financial and ethical frameworks posed a challenge to implementation of such solutions [83].

Acceptance was higher when efforts were made by design researchers to orient and explain to future users. To study the acceptance of assistant robots in the living arrangement of older adults, researchers made initial efforts to broadly communicate information about Robot-Era Experimentation in an Italian municipality, before making a general call for volunteers (n=35). Participants were offered experience with three different robotic systems, tested in various locations (apartment, house, outdoors), thus were provided an opportunity to explore and become interested in different affordances of the technology in assisting with every-day living tasks [84].

# Testing A-ICTs for Early Detection of Risks Associated with Age-Deficit

The risks associated with ageing were the rationale for developing risk-preventative technologies. Health monitoring and ecological research were also among reasons for suggesting healthy ageing through prevention.

A multinational RCT of internet-based counselling in older adults in higher risk of cardiovascular disease was tested among a large sample (>2700 participants across France, Netherlands and Finland) and proved to generate modest improvements in markers such as blood pressure, cholesterol and body mass index, over the 18 month, with improvements slightly larger in the intervention group [85]. A secondary analysis of this data revealed that degree of engagement with the system (higher in those with higher digital literacy) was associated with the larger benefits [86].

*"With the ultimate aim of early diagnosis of dementia"*, a VR-based body balance assessment system was developed, and validated by testing the hypothesis that compared to young adults (n-15), healthy older adults (n=14) would sway more in anterior-posterior (AP) direction, thus suggesting that the system could be used to detect differences between healthy older adults and those in risk of developing dementia [87].

Based on assumption that older adults are at greater risk for impaired driving and road accidents, a machine learning algorithm was developed that incorporated various 'clinical' factor including age, to predict safe and unsafe drivers. The accuracy of the classifier was tested in (n=33) healthy older adults, whose driving behaviors were ecologically monitored using video and speed recording while they drove [88].

Addressing the stress of informal caregiving to older adults with dementia, a multinational project *"intelligent system for independent living and self-care of seniors with cognitive problems or mild dementia"* aimed to test the impact of integrating automated and interactive A-ICTs in the lives of older adults. Over 15 months older adults with CI and their informal caregivers (ICGs) from four European pilot sites (Denmark, Finland, Greece, and UK), were followed up and it was shown that in the intervention group (n=45) to be able to receive notifications (about risk or needs) of older adults was beneficial to the quality of life of the caregivers; however the heterogeneity in uptake and beneficence among regions and individuals led to the conclusion that an individualized approach to addressing the needs of persons with dementia was essential [89].

# Future Role of A-ICTs in Health Care for Ageing Populations

Various multi-stakeholder studies examined the barriers to adoption of A-ICTs, against the background of envisioning for the growing cost of care for an ageing population.

A multi-stakeholder study involving ICT instructors (35 occupation therapy students), well-educated and computer-owning older ICT trainees (n=12) and community care-providers to older adults (n=14) revealed that digital literacy is not the only barrier to adoption of home-based ICTs for chronic health conditions. Rather personal as well as socio-contextual factors contribute to whether target users find the interventions meaningful to adopt [90].

In a relatively large-scale qualitative study involving 27 focus groups comprising of grandmothers who use internet (in Canada, Columbia, Israel, Italy, Peru, Romania and Spain), it was shown that older adults intentionally limit their usage, and use health-related ICTs depending on the circumstances and needs arising from their age-related conditions such as spousal loss, physical constraints and retirement limiting their social circles [91].

In an Italian pilot study within a multinational project for *Healthy and Active Ageing*, older adults (n=22), informal caregivers (n=22), professional caregivers (not specified whether they were doctors or nurses, but they had access to health data, n=13) and social operators (n=4) were interviewed to identify the stakeholder’s needs, and by discussing various possible technologies (Internet or Things, Apps, wearable sensors, Artificial Intelligence, Robots and augmented and virtual reality) determined two fundamental need categories: Heath Management (i.e., stimulation and monitoring), and Socialization (i.e., promoting social inclusion) [92].

In another Italian study within a multinational project for "Safety of Elderly People and Vicinity Ensuring" involving installation of home monitoring systems (to signal flooding, gas leak, control temperature, communicate via teleconferencing, receive telemedicine and be reminded of to-do lists), a qualitative analysis of the perspective of older adults (n=13), informal caregivers (n=8) and other stakeholders such as end-user representatives, and psychology researchers (n=9), showed that the usefulness of these technologies was tied to learning facility, and ability to operate and control them actively and independently [93].

In another multi-stakeholder Focus group study in the Netherlands, focusing on deploying technologies to promote ageing in place, fine parallel focus groups consisting of older adults (n=6), homecare professionals (providing care themselves or coordinating non-family caregivers) n=7), managers in a homecare or social work organizations (n=5), technology designers (n=6) and policymakers (n=5) revealed that from a range of 26 possible technologies designed for promoting independent living among older adults, only 6 were mentioned by all (health monitoring, assistive technology, home automation, household appliances, computers, and video telephony) while lifestyle monitoring, lift assist devices, and global positioning system (GPS) navigation were mentioned by technologists only. Tailoring technology to the specific needs of each community-dwelling older adults was the most critical factor for successful adoption of technologies [94].

A qualitative year-long study in Switzerland, an ambient home monitoring sensor system in conjunction with a wearable biosensor was evaluated by home dwelling older adults (n=13), family caregivers (n=13) and nurses (n=20). This study showed greater enthusiasm about the benefits of these systems among older adults and family caregivers, and concerns among nurses to lose contact with older adults. It is noteworthy, however, that 42.5% of those who passed eligibility screening (127 out of 192 initially targeted older adults) refused to participate [95].

Lack of knowledge about the affordances of technology were found in a German Focus Group study of the challenges of adopting assistive technologies. In this study Participants (n=11 community dwelling older adults recruited from a list of geriatric care hospital) were guided to speak about wishes and worries of three older persona with psychological, physical and social deficits likely in old age, suggest possibilities of supporting them, and identify requirements for successful use of assistive devices. Discussions indicated that the largest obstacle to use of technology was lack of knowledge about their existence and affordance [97].

To envision future ICTs to contribute to the community healthcare in a European project, designers conducted a multi-stakeholder focus group study with older adults (n=1), family caregivers (n=14) and professionals such as healthcare managers (n=15) in two countries (Slovenia and Switzerland), and concluded that despite openness, ICT use and familiarity was too low to generate customizable solutions [96].
